# Supplementary material for: A plastid two-pore channel essential for inter-organelle communication and growth of Toxoplasma gondii
Source: Nat Commun. 2021 Oct 4;12:5802. doi: 10.1038/s41467-021-25987-5 (PMC8490419; doi:10.1038/s41467-021-25987-5)
Supplement: Supplementary file 1 — Supplementary Information [file 41467_2021_25987_MOESM1_ESM.pdf]

A plastid Two-Pore Channel essential for inter-organelle communication and growth of *Toxoplasma gondii*

Li, Z. et al

Supplementary Information

Supplementary Table 1. Sequences used for phylogenetic analysis

Abbreviation	Species	Accession Numbers
TgTPC	*Toxoplasma gondii*	TGGT1_311080
EtTPC	*Eimeria tenella*	XP_013233675.1
EnTPC	*Eimeria necatrix*	XP_013434114.1
NcTPC	*Neospora caninum Liverpool*	XP_003885109
HhTPC	*Hammondia hammondi strain H.H.34*	XP_008885893
BbTPC	*Besnoitia besnoiti*	PFH34105.1
CsTPC	*Cystoisospora suis*	PHJ18043.1
VbTPCa	*Vitrella brassicaformis*	CEL98608.1
VbTPCb	*Vitrella brassicaformis*	CEL93554.1
AtTPC	*Arabidopsis thaliana*	NP_567258.1
OsTPC	*Oryza sativa Japonica group*	XP_015621659.1
BnaTPC	*Brassica napus*	CDY21975.1
TtTPCRd	*Thecamonas trahens*	AMSG_09817
TtTPCRa	*Thecamonas trahens*	AMSG_03223
CoTPC1	*Capsaspora owczarzaki*	EFW46182
SrTPC1b	*Salpingoeca sp. ATCC 50818*	EGD80440
SrTPC2	*Salpingoeca sp. ATCC 50818*	EGD77972
MbTPC1a	*Monosiga brevicollis*	XP_001750879.1
MbTPC1b	*Monosiga brevicollis*	XP_001742806
MbTPC3	*Monosiga brevicollis*	XP_001744080
MbTPCRb	*Monosiga brevicollis*	XP_001749979.1
SpTPC1	*Strongylocentrotus purpuratus*	CBI63263.1
SpTPC2	*Strongylocentrotus purpuratus*	CBI63264.1
SpTPC3	*Strongylocentrotus purpuratus*	CBI63265.1
HsTPC1	*Homo sapiens*	NP_001137291.1
HsTPC2	*Homo sapiens*	NP_620714.2

Supplementary Table 2: Cell lines created in this study

Cell lines	Parental	Description	3' tagging of TgTPC	Drug selection
iΔTPC	*TatiΔku80*	5' inducible T7S4 promoter insertion	-	pyrimethamine
TgTPC-3HA	*TatiΔku80*	3' 3HA tagging	HA	chloramphenicol
iΔTPC-3HA	*TatiΔku80*	5' inducible T7S4 promoter insertion and 3' 3HA tagging	HA	pyrimethamine + chloramphenicol
iΔTPC-TR	*iΔTPC*	iΔTPC resistant to ATc	-	pyrimethamine
iΔTPC-3HA-TR	*iΔTPC-3HA*	iΔTPC-3HA resistant to ATc	HA	pyrimethamine + chloramphenicol
*ΔTPC (ΔTPC-a is a subclone)**	*iΔTPC*-TR	Knockout of TgTPC (5 kb of TPC gDNA was deleted)	-	chloramphenicol
ΔTPC-a-TPC	*ΔTPC-a*	ΔTPC-a complemented with TgTPC cDNA under Tubulin promoter	Myc	chloramphenicol + pyrimethamine
ΔTPC-a-iTPC	*ΔTPC-a*	ΔTPC-a complemented with TgTPC cDNA under inducible T7S4 promoter	Myc	chloramphenicol + pyrimethamine
ΔTPC-a-iTPC[*1]	*ΔTPC-a*	ΔTPC-a complemented with TgTPC cDNA with pore 1 mutation under inducible T7S4 promoter	Myc	chloramphenicol + pyrimethamine
ΔTPC-a-iTPC[*2]	*ΔTPC-a*	ΔTPC-a complemented with TgTPC cDNA with pore 2 mutation under inducible T7S4 promoter	Myc	chloramphenicol + pyrimethamine
RH-FNR-GCaMP6-HA	RH	FNR-GCaMP6-HA transfected into RH cells	-	pyrimethamine
ΔTPC-a-FNR-GCaMP6-HA	*ΔTPC-a*	FNR-GCaMP6-HA transfected into ΔTPC-a	-	chloramphenicol + pyrimethamine
ΔTPC-a-TPC-FNR-GCaMP6-HA	*ΔTPC-a-FNR-GCaMP6-HA*	*ΔTPC-a-FNR-GCaMP6-HA* complemented with TgTPC cDNA under Tubulin promoter	Myc	chloramphenicol + pyrimethamine

***Generation of the ΔTPC-a cells.**

We transfected *iΔTPC* cells with the tdTomato (tandem dimer fluorescent protein) gene (*doi: 10.1038/nbt1037*) and isolated red clones by FACS sorting. tdTomato expressing cells were used to infect fibroblast cells in 32 mm dishes. Cultures were propagated in media with 0.5 µg/ml ATc. Plaques were only formed if the media was changed every 4 days and they were very small and only visible 25 days after the initial inoculation. We fixed and stained plaques at this time for quantification purposes. We estimated that 1 out of 7,100 *iΔTPC* cells initially inoculated were able to form small plaques 25 days p.i. in the presence of ATc. Similar results were obtained with other *iΔTPC* cell lines. Based on this result, we reasoned that very few *iΔTPC* cells were able to grow in the presence of ATc at a very slow rate. *iΔTPC (+ATc)* cells were unable to lyse host cells at this growth rate and host cells started to age and detach at around 25 days. Because of this, we manually broke the cultures by scrapping off infected monolayers passing them through a needle to release parasites. We performed this manual passage every 7 days. At each passage, manually released parasites were allowed to infect fresh host cells and grown in the presence of ATc. Cells were manually passed for a minimum of 6 weeks at which point the parasites were able to lyse host cells on their own. This process generated the TR cell lines that were obtained from the *iΔTPC*, *iΔTPC-3HA* and *iΔTPC-RFP* lines. Western blots were performed for possible leaky expression of TPC-3HA in the iΔTPC-3HA-TR cell line. No TPC-3HA was detected in two separate iΔTPC-3HA-TR cell lines, confirming that their capacity to grow in the presence of ATc was not because of leaky expression of *TgTPC*.

Supplementary Table 3: Whole genome sequencing of TPC mutants*

Chromosome #	Position	TGGT1	iΔTPC	ΔTPC-a	ΔTPC-b	ΔTPC-c-E5	iΔTPC-TR
TGGT1_Ia	327582	CATAT	CATAT	C	-	C	C
TGGT1_Ia	637221	G	G	GAT	GAT	GAT	G;GAT
TGGT1_Ia	1035474	G	G	GTATATATATA	GTATATATA	GTATATATA	GTATATATA
TGGT1_Ib	510631	A	A	AATATAT	-	AATATAT	-
TGGT1_Ib	788668	CATATATAT	CATATATAT	-	-	-	C
TGGT1_II	41993	T	T	TTATA	-	-	-
TGGT1_II	141891	TTA	TTA	T	T	T	T
TGGT1_II	342152	AAT	AAT	A	-	A	-
TGGT1_II	647531	AAT	AAT	-	-	-	A
TGGT1_III	1944898	CATATATAT	CATATATAT	-	-	-	C
TGGT1_V	642264	CATATATATAT	CATATATATAT	-	-	C	C
TGGT1_V	1522040	CTATA	CTATA	C	-	CTATA	-
TGGT1_VI	1717559	GATATATAT	GATATATAT	-	-	-	G
TGGT1_VI	3432502	TATAGATAG	TATAGATAG	T	-	T	T
TGGT1_VIIa	1695582	TTATATATATATA	TTATATATATATA	-	T	T	T
TGGT1_VIIa	1890137	CATATAT	CATATAT	-	-	C	-
TGGT1_VIIb	1910002	AATATAT	AATATAT	A	-	A	-
TGGT1_VIIb	2041732	GCT	GCT	-	-	GCT	G
TGGT1_VIIb	2431339	GTATATATA	GTATATATA	G	-	G	-
TGGT1_VIII	1124400	CTCTTCT	CTCTTCT	C	C	C	C
TGGT1_IX	2296623	AATATAT	AATATAT	A	A	A	A
TGGT1_IX	2307530	TATATAG	TATATAG	T	T	TATATAG	-
TGGT1_XI	4928189	ATATC	ATATC	A	-	A	A
TGGT1_XII	3195311	TATAG	TATAG	-	-	-	T

Table lists nucleotide differences (indels) that appeared in at least 3 genomes out of the 4 mutants (*ΔTPC-a, ΔTPC-b, ΔTPC-c5, and iΔTPC-TR*) sequenced. Each genome was compared with the reference strain TgGT1 and with the parental strain iΔTPC. If the indel was present in only one or 2 of the mutant genomes we assumed that it was not a relevant change. All these indels are in intergenic region.

*NCBI Accession: PRJNA695295 or https://www.ncbi.nlm.nih.gov/sra/PRJNA695295

Supplementary Table 4: Primers used in this study

Primer #	Sequence (underlined nucleotides correspond to restriction sites)
1	CCGAGATCTATGGCGCTTAACAACGGTAGT
2	CGACCTAGGGAGCACAGGACGCCAAGAACT
3	TACTTCCAATCCAATTTAATGCCAGGCAGTGCGTCGGCGTTC
4	TCCTCCACTTCCAATTTTAGCGAGCACAGGACGCCAAGAACTG
5	CCGCATATGCCAGGCGTAGACACTAGCAAAC
6	CCGCATATGGGTCGGTAAAAAGAGGAGAGAGA
7	CGACCTAGGGTAGCGTGAAGAAAGCGCGA
8	CGTCTGGAGGCGACTTGCGCCGAGCAGCTGCATGTTGGGTGTACAGC GTGCCTCGACTACGGCTTCCATTGGCAAC
9	CTGACCAGACAGGCAGACGGCCTTTCCTGTGAAGTTCGAACTTGCTGC TGCATACGACTCACTATAGGGCGAATTGG
10	CGACCTAGGGTAGCGTGAAGAAAGCGCGA
11	AAACGCGCCGGAAAACGCGCCT
12	GGCGAGCCTTGACGCTCCTGTGAA
13	GAGCGGCTGTATTTCGCCGCTTCTCGTCGTCACAAC
14	GTTGTGACGACGAGAAGCGGCGAAATACAGCCGCTC
15	GCCTCACTCGTCACGCCATTTCTTCTCACTGTCAAC
16	GTTGACAGTGAGAAGAAATGGCGTGACGAGTGAGGC
17	CTTCGTTACCGTGATGCAAA
18	ATGACGCGAAAGACCTGAAT
19	TGGGTTGAGGTCCCTCATTA
20	TCTATTGCAATGGAAAAAGGTATG
21	TCAATGGTAGAGCAAAGGACTG
22	AGATCTATGGTTCGGGGCATCCGTCCTC
23	TGAGAACCCATGGATGTTTGGTCGGTCGGGGT
24	CCAAACATCCATGGGTTCTCATCATCATCATC
25	CCTAGGCTTCGCTGTCATCATTTGTACA

Supplemental Figures and Legends:

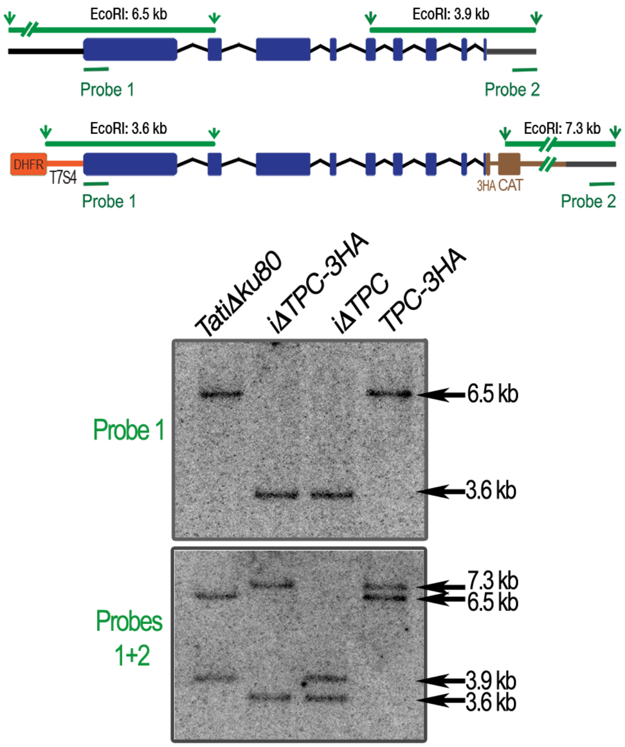

Supplementary Figure 1: Southern blot analysis of genomic DNA isolated from *iΔTPC-3HA* and *TPC-3HA* mutants. The triple HA tag was inserted at the 3' end of the genomic locus of the *TgTPC gene*. The DHFR-T7S4 cassette was inserted at the 5' end of the *TgTPC gene* to create the *iΔTPC* and *iΔTPC-3HA* mutants. Genomic DNA was purified and digested with EcoRI. The membrane was first hybridized with probe 1 that targets the 5' region of the coding sequences, then the same membrane was used for hybridization with probe 2, which targets the 3' UTR of the *TgTPC* gene without washing off probe 1. Promoter inserted parasites (*iΔTPC)* showed a shift of the band hybridized to probe 1 (from 6.5 kb in wild type cells to 3.6 kb in promoter inserted cells). The 3' tagged parasites (*TPC-3HA*) showed a modified band hybridized to probe 2 (from 3.9 kb in wild type cells to 7.3 kb in 3'-tagged cells). In parasites that were modified at both ends, *iΔTPC-3HA*, the sizes of both bands were changed. $N = 2$

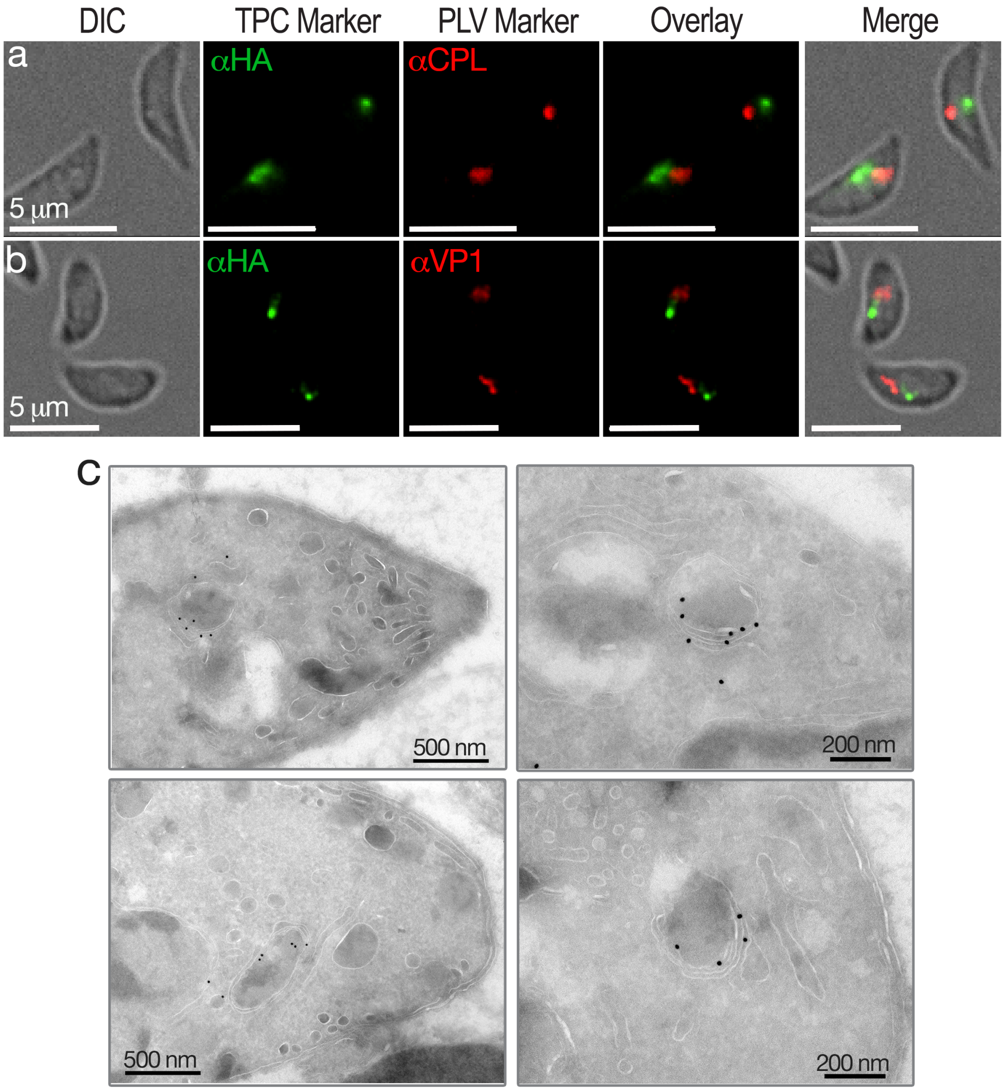

Supplementary Figure 2: The TgTPC-3HA signal does not localize to the plant-like vacuole. IFAs of extracellular tachyzoites of the *TgTPC-3HA* mutant. **a**, Parasites were purified and fixed as detailed in the Methods section and were labeled with the rabbit anti-TgCPL (1:1000, *red*) and αHA (1:200, green). *N* = 3. **b**, co-localization of the αHA (1:200, *green*) signal with Guinea pig anti-TgVP1 (1:400, *red*). *N* = 3. **c**, ImmunoEM of TgTPC-3HA tachyzoites showing the specificity of the gold labeling in the apicoplast which is identified by its multiple membranes. *N* = 2

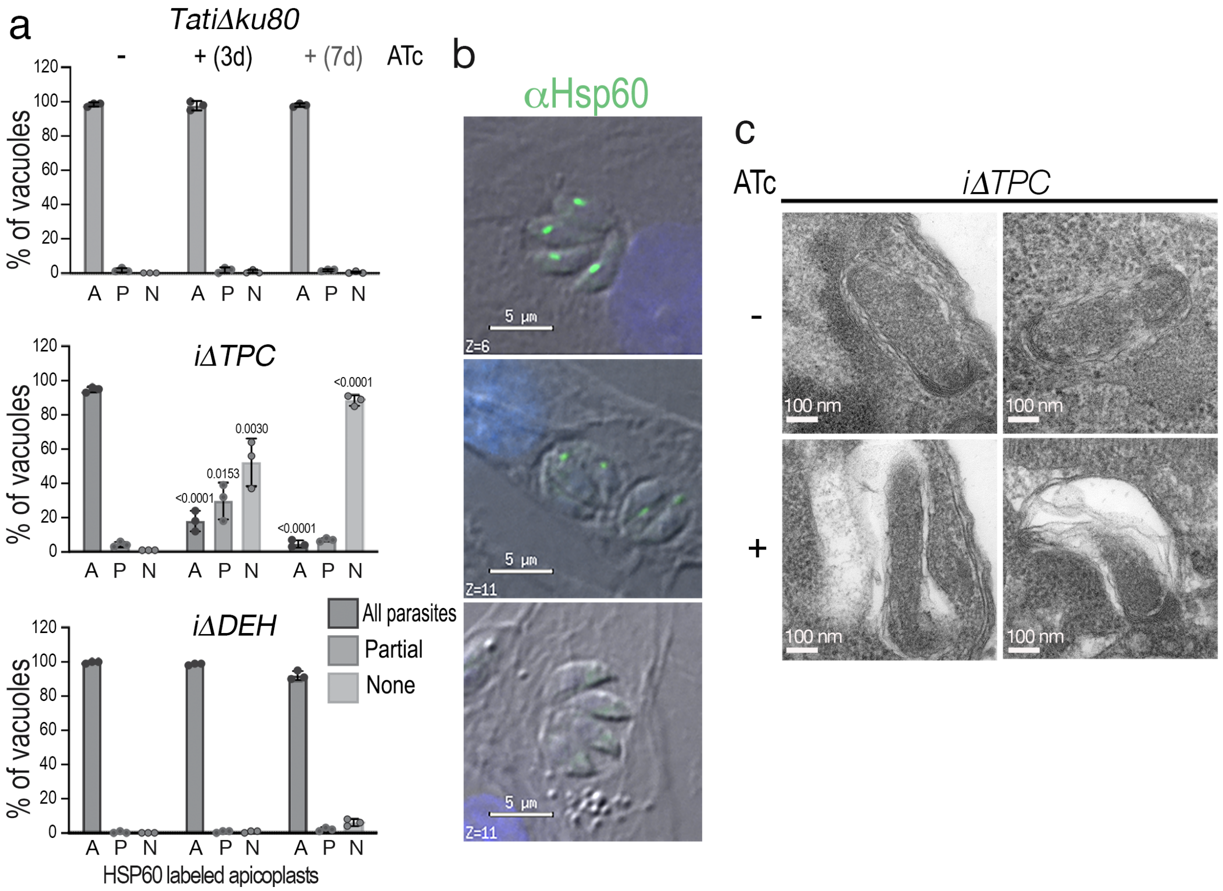

Supplementary Figure 3: Apicoplast integrity of the *iΔTPC-3HA* mutants cultured with ATc. a, Quantification of parasites with and without intact apicoplast after culturing them with ATc. *Hsp60* expression was used for the quantification. The conditional mutants for *iΔDEH* (hydroxyacyl-CoA dehydratase), which are also responsive to tetracycline but in which the apicoplasts are not affected is also shown (*Ramakrishnan S. et al, Mol. Microbiol. 2015* ***97****, 64-76*). $N = 3$. p values: unpaired two tailed t test performed to compare A, P and N of ATc treated parasites with untreated. **A:** all parasites inside parasitophorous vacuoles (PVs) show intact apicoplasts, **P:** only a fraction of the parasites in PVs shows apicoplast labeling, **N:** None of the parasites inside PVs show apicoplast labeling. Results show mean ± SD**. b,** examples of All, Partial or None of the parasites having an intact apicoplast in one parasitophorous vacuole. $N = 3$. **c**, Transmission electron microscopy images of the *iΔTPC* mutant cultured with and without ATc for four days showing normal (-ATc) and misshaped apicoplasts (+ATc). $N = 2$.

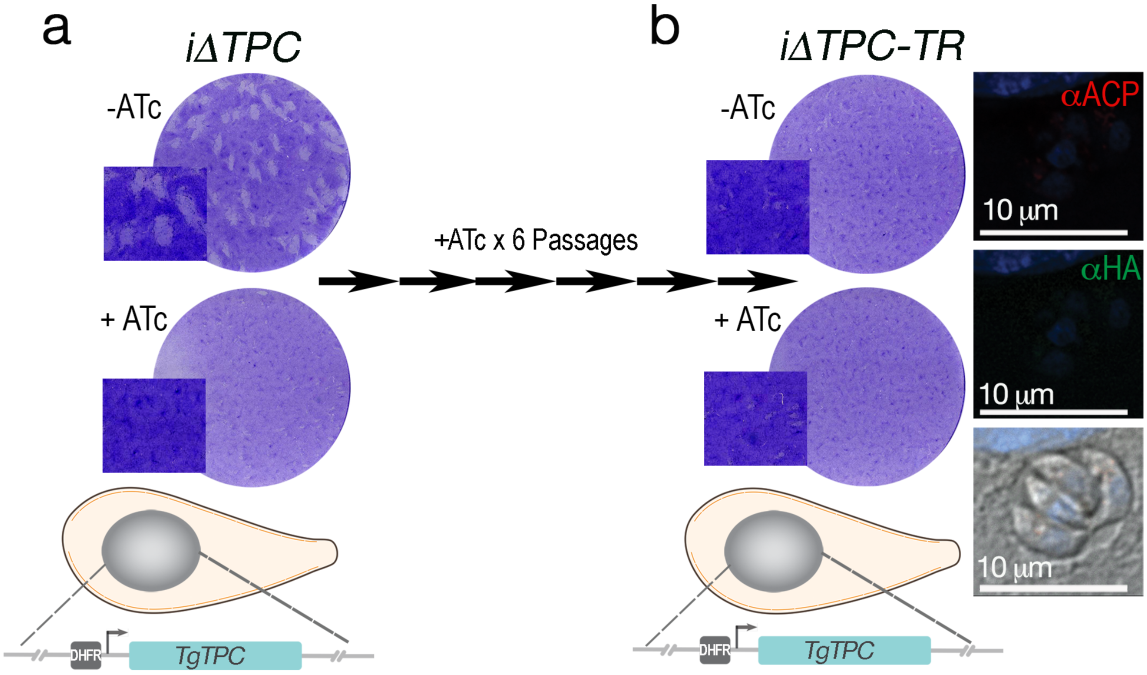

Supplementary Figure 4: Isolation of *ΔTPC* mutants. **a,** The promoter inserted *iΔTPC* parasites were passed manually through syringe and used to re-infect host cells. *iΔTPC-TR* cells were isolated after 6 weeks (one passage per week). **b**, *iΔTPC-TR* parasites are able to grow without manually breaking host cells. However, they only form small plaques after 13 days of incubation. Plaque sizes were not affected by the addition of ATc. $N = 3$. Detailed protocol is described under Supplementary Table 2.

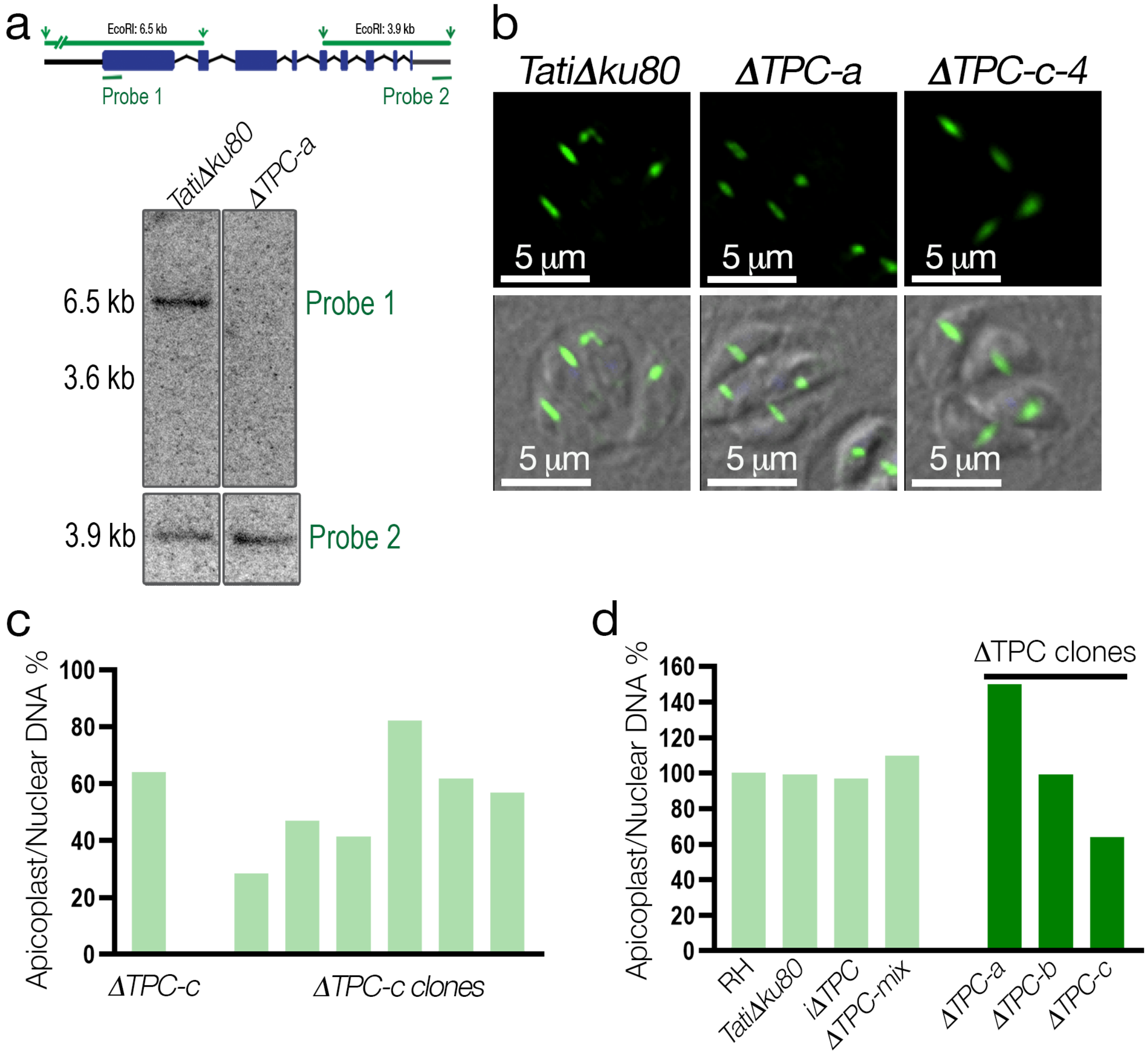

Supplementary Figure 5: Southern blots and apicoplast DNA of the *ΔTPC* mutant. **a,** Southern blot analysis showing that the 5' coding sequence is absent in the *ΔTPC* KO mutant. The membrane was first hybridized to probe 1, which results in the 6.5 kb band shown. This is the same probe used for the Southern shown in Fig. S1 against the 5'-ORF of TPC. The same membrane was then hybridized to probe 2. This hybridization was also considered as loading control. The 3'-UTR (the 3.9 kb band resulting from hybridization to probe 2) is still present. *N* = 2. **b,** knockout clones termed *ΔTPC-c* were subcloned and grown for 5 months and they showed apicoplast labeling by IFA with anti-Hsp60 (1:1,000). One subclone (*ΔTPC-c-4*), and *TatiΔKu80* and *ΔTPC-a* clones are shown for comparison. *N* = 3. **c,** apicoplast DNA from *ΔTPC-c* clone and subclones determined by Southern blot analysis with both nuclear and apicoplast DNA probes. **d,** TPC *null* mutant clones appear to have variable amounts of apicoplast DNA copy numbers, compared to control cell lines RH and TatiΔku80 and the conditional mutant without ATc (*iΔTPC*).

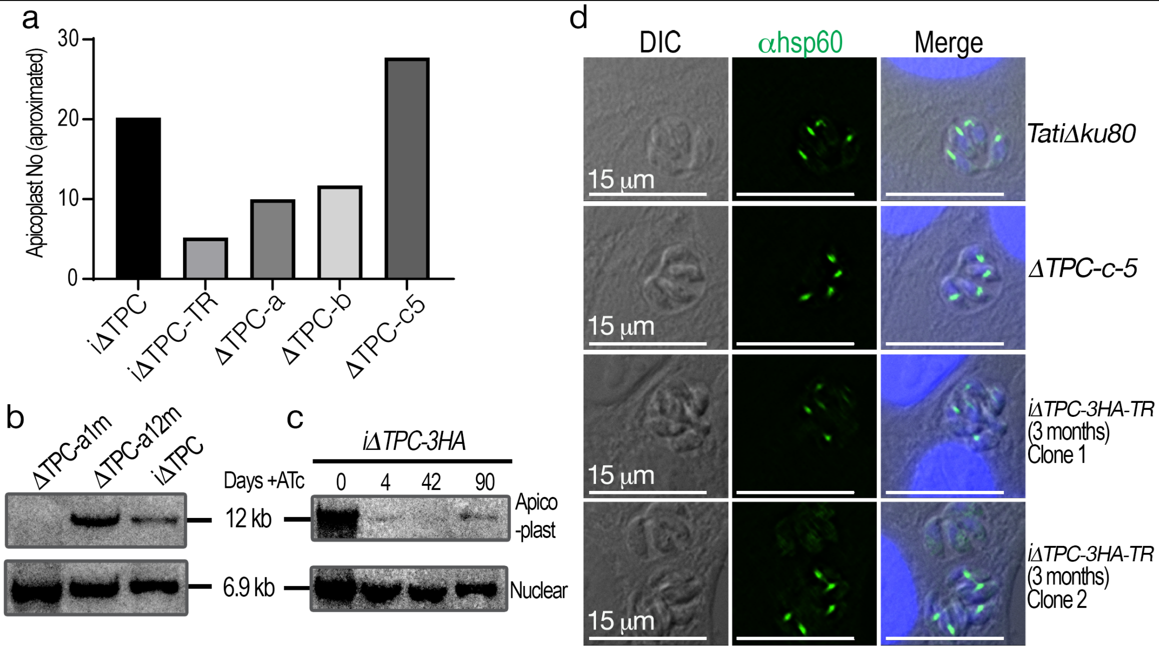

Supplementary Figure 6: Increase in apicoplast labeling correlates with higher levels of apicoplast DNA. **a**, estimated apicoplast DNA copy #s in the cells lines that had their whole genome sequenced (Table S3). The estimated apicoplast DNA copy number was calculated as coverage of apicoplast genome/coverage of nuclear genome assuming that all the DNA fragments in nuclear and apicoplast DNA are equally amplified and sequenced. Note that the *ΔTPC-c5* mutant with the highest apicoplast DNA also showed that all cells were labeled with the apicoplast marker hsp60 in **d**. **b**, Increase of apicoplast DNA in *ΔTPC* cells with time in culture. The total DNA extracted from each cell line was digested with EcoRI, then hybridized to a radiolabeled apicoplast DNA probe and a nuclear DNA probe. *ΔTPC-a1m*: *ΔTPC-a* cells cultured *in vitro* for one month. *ΔTPC-a12m*: *ΔTPC-a* cells cultured *in vitro* for 12 months. $N = 2$. **c**, Apicoplast DNA increase with time in culture of the *iΔTPC-3HA-TR* mutant. The *iΔTPC-3HA* treated with ATc for 42 and 90 days are the TR cell lines. $N = 2$. **d**, Higher apicoplast IFA signal in TR cells after 3 months of culture *in vitro*. $N = 3$

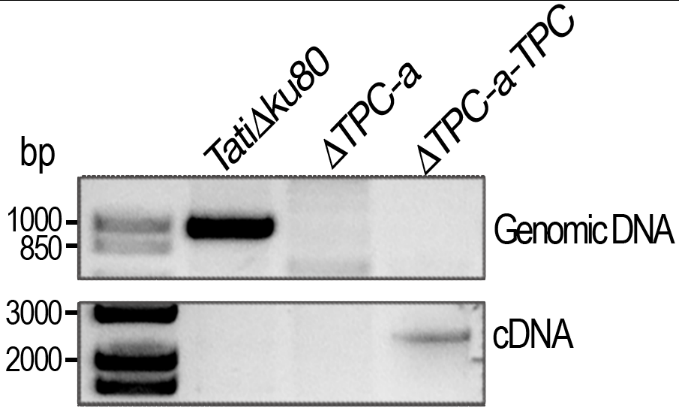

Supplementary Figure 7: PCR showing the presence of *TgTPC cDNA* in the *ΔTPC-a-TPC* mutant. PCR with primers 1 and 10 (Supplementary Table 4) amplified a 2.4 kb cDNA fragment. These two primers failed to amplify *T. gondii* genomic DNA because of the presence of introns. Primers 11 and 12 (Supplementary Table 4) were used to amplify the wild type TPC locus. $N = 3$.

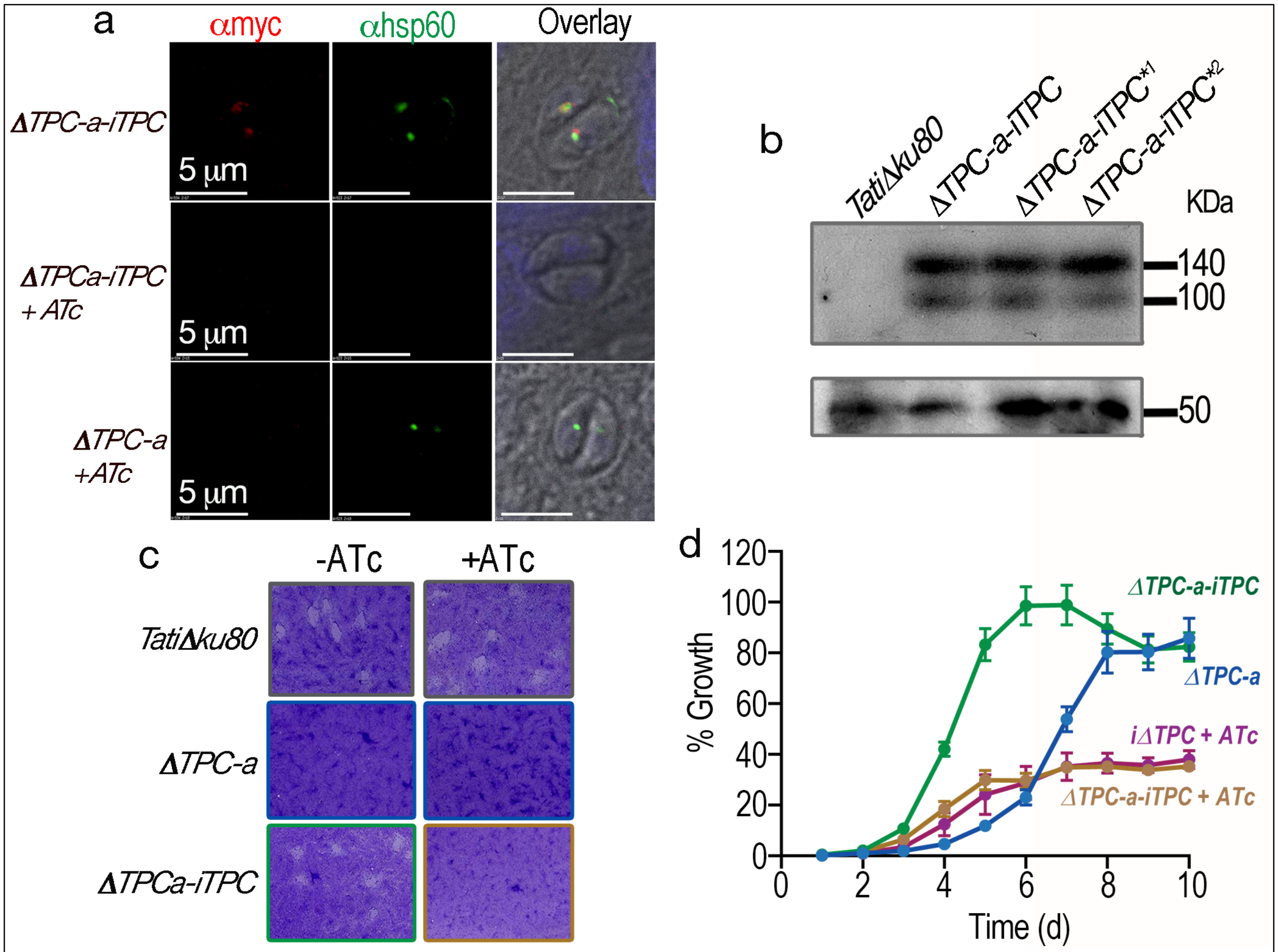

Supplementary Figure 8: Complementation of *ΔTPC-a* cells with a conditionally expressed copy of *TgTPC* (*ΔTPC-a-iTPC*). a, Immunofluorescence of cells expressing TgTPC-cMyc with αcMyc showing co-localization of TgTPC with the apicoplast marker Hsp60. Addition of ATc leads to disappearance of both apicoplast and TgTPC-myc signals (*ΔTPCa-iTPC+ATc). N* = 3. **b**, Western blots with αMyc antibody confirming expression of TgTPC in the conditionally complemented mutants. The lower panel show loading control with α-tubulin. *N* = 1. **c**, Plaque assays of *TatiΔku80*, *ΔTPC-a* and *ΔTPCa-iTPC* with and without ATc. *N* = 3. **d**, Growth of *ΔTPC-a-iTPC* and *ΔTPC-a*, without ATC compared to growth of *iΔTPC* and *ΔTPC-a-iTPC* with ATc. 4,000 tdTomato expressing parasites were added per well. In the absence of ATc, the conditionally complemented cells *ΔTPC-a-iTPC* (*green*) show normal growth. In the presence of ATc, the growth of the conditionally complemented *ΔTPC-a-iTPC* mutant was inhibited. A standard curve for each cell line was developed for fluorescence vs. number of parasites. *N* = 3.

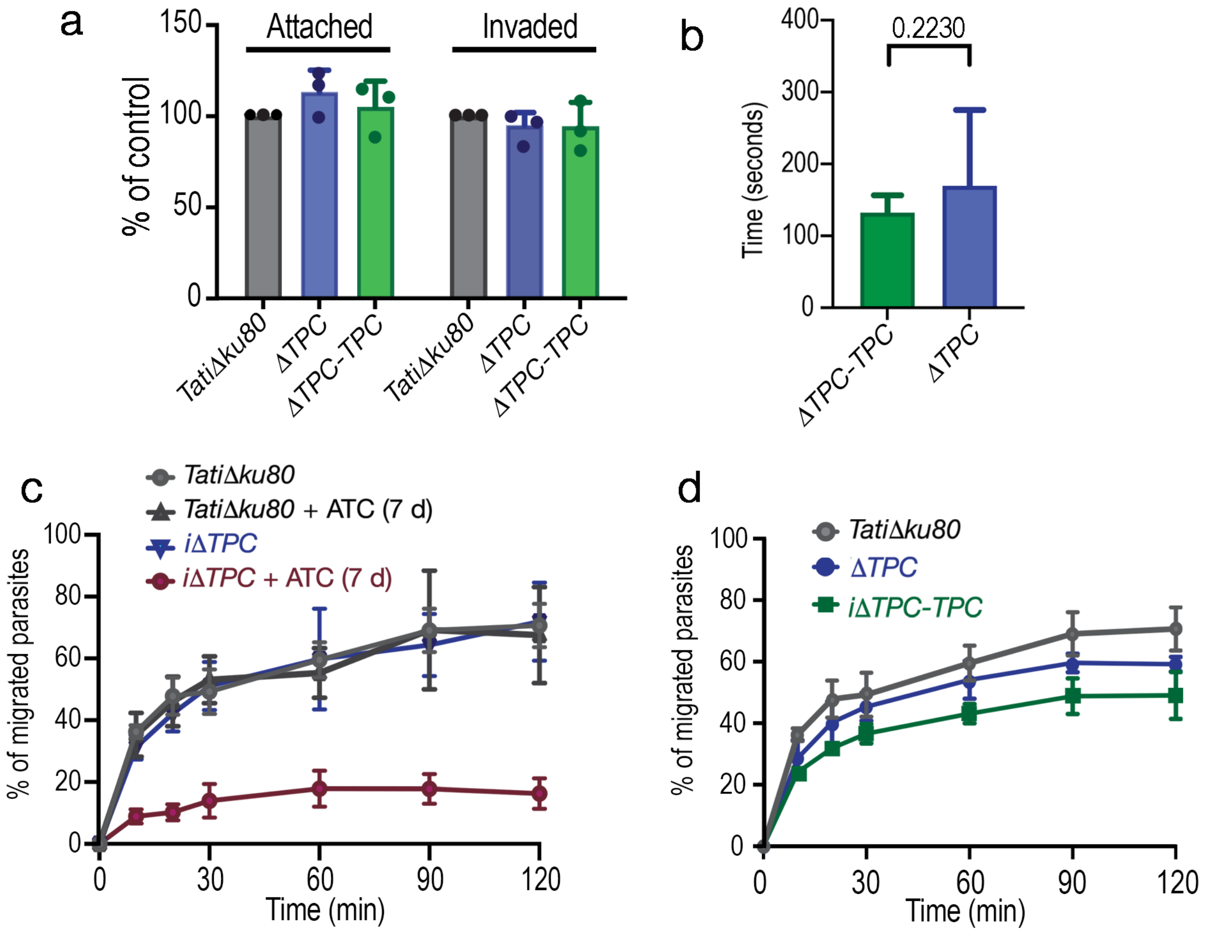

Supplementary Figure 9: Lytic cycle characterization of *ΔTPC* mutants. a, Red-green assay to evaluate invasion and attachment of *ΔTPC-a, TatiΔku80* and *ΔTPC-a-ΔTPC* lines. $N = 3$. **b**, Time to egress of infected cells triggered with 1 µM ionomycin. No statistical significance between time to egress of *ΔTPC-a* and *ΔTPC-a-TPC* mutants ($p = 0.2230$, n = 13, $N = 4$). **c**, Transwell migration through a 3 µm pore membrane. Parasites that migrate to the lower compartment were counted at the indicated times. *iΔTPC+ATc (magenta)* showed a significant difference compared to the parental line *TatiΔku80* (with and without ATc) and the same mutant without ATc. Values are means ± SD ($p = 0.0153$, at 90 min, *iΔTPC* vs *TatiΔku80*, both previously cultured with ATc for 7 days). $N = 3$. **d**, Transwell migration through a 3 µm pore membrane. Parasites that migrate to the lower compartment were counted at different times. *ΔTPC* (blue) migrate at similar rate than the parental lines ($p = 0.2324$, *ΔTPC* vs *TatiΔku80*, 90 min time point). $N = 3$. p value: unpaired two tailed t test performed for all comparisons.

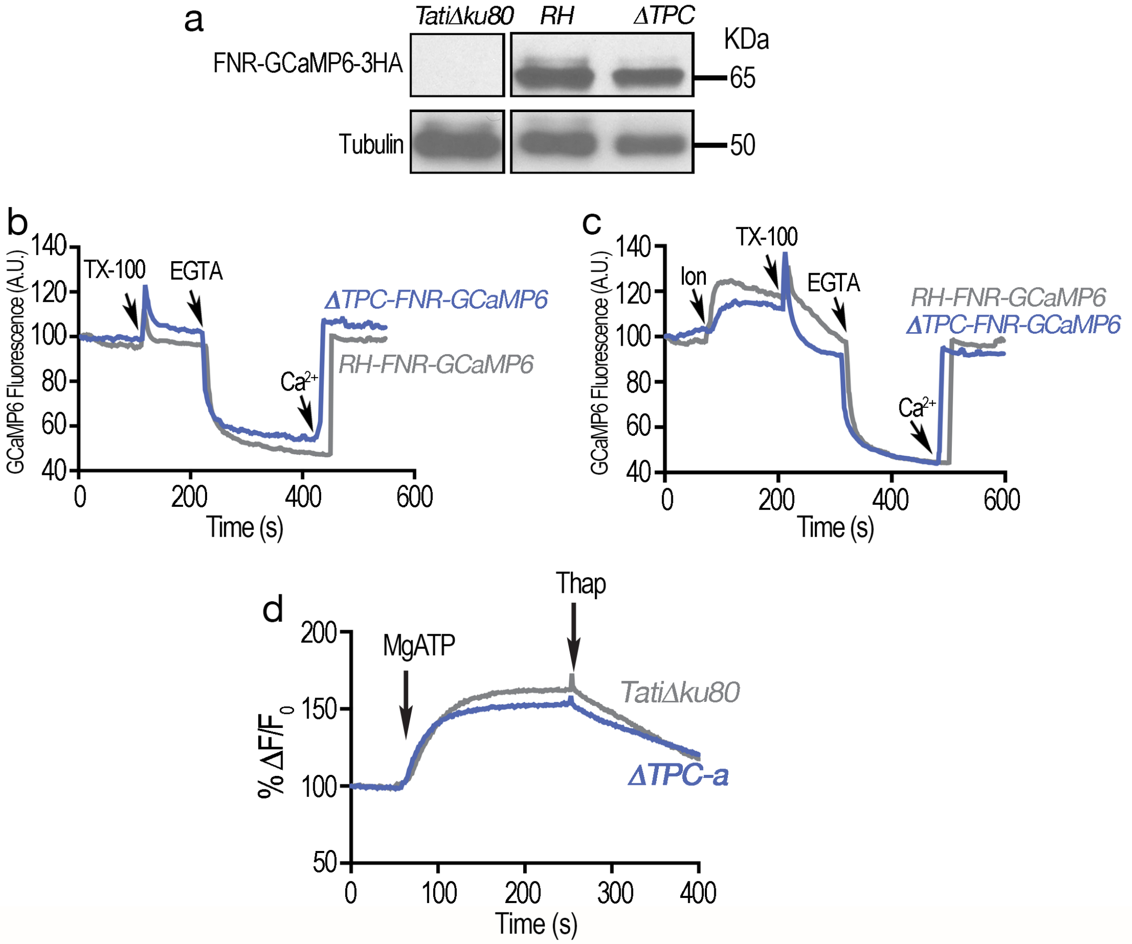

Supplementary Figure 10. Expression of a Genetically Encoded Ca^{2+} indicator in the apicoplast. **a**, Western blot analysis of total lysates from *TatiΔku80*, *ΔTPC-FNR-GCaMP6* and *RH-FNR-GCaMP6* parasites with mouse anti-HA (1:1,000) showing similar expression levels of the GCaMP6 indicator. $N = 2$. **b**, Control experiments with suspension of parasites expressing GCaMP6 and measuring fluorescence of the suspension. The experiments show the response of the fluorescence to EGTA (100 µM) and Ca^{2+} (1.8 mM) after adding 0.1% Triton X-100 for permeabilization and exposure of the indicator to the added reagents. **c**, similar control but adding ionomycin (Ion) prior to the detergent. **d**, *ΔTPC-a* tachyzoites were loaded with MagFluo4-AM for organellar compartmentalization. Addition of MgATP, the substrate of the SERCA-Ca^{2+}-ATPase leads to an increase in fluorescence indicating Ca^{2+} uptake into the ER. Addition of thapsigargin (Thap) results in leakage of Ca^{2+}. The experiment shows that the uptake activity is similar in the *ΔTPC-a* to the control strain. $N = 3$.

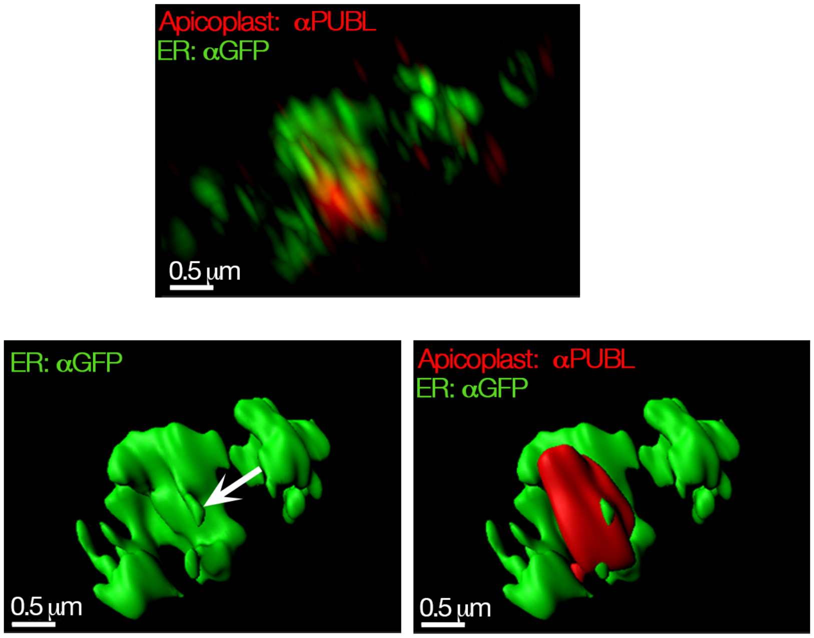

Supplementary Figure 11: Super resolution microscopy and 3D reconstruction showing close interaction between the apicoplast (*red)* and the endoplasmic reticulum (*green*). RH cells were transiently transfected with a plasmid encoding a gene for P30-GFP-HDEL, which has been shown to selectively localize to the ER. The GFP was labeled using an anti-GFP antibody at a 1:100 dilution. The apicoplast was labeled using a 1:200 dilution of an anti-PUBL antibody (*Fellows, JD, et al, 2017, MBio 8*). PUBL is a protein that has been shown to localize to the apicoplast. *N* = 2.
