# Supplementary material for: A plastid two-pore channel essential for inter-organelle communication and growth of Toxoplasma gondii
Source: Nat Commun. 2021 Oct 4;12:5802. doi: 10.1038/s41467-021-25987-5 (PMC8490419; doi:10.1038/s41467-021-25987-5)
Supplement: Supplementary file 3 — Reporting Summary [file 41467_2021_25987_MOESM3_ESM.pdf]

nature portfolio

Corresponding author(s): DBPR, Manuscript NCOMMS-21-04332-T

Last updated by author(s): Aug 26, 2021

Reporting Summary

Nature Portfolio wishes to improve the reproducibility of the work that we publish. This form provides structure for consistency and transparency in reporting. For further information on Nature Portfolio policies, see our Editorial Policies and the Editorial Policy Checklist.

Statistics

For all statistical analyses, confirm that the following items are present in the figure legend, table legend, main text, or Methods section.

n/a	Confirmed	
☐	☒	The exact sample size (n) for each experimental group/condition, given as a discrete number and unit of measurement
☐	☒	A statement on whether measurements were taken from distinct samples or whether the same sample was measured repeatedly
☐	☒	The statistical test(s) used AND whether they are one- or two-sided<br>*Only common tests should be described solely by name; describe more complex techniques in the Methods section.*
☒	☐	A description of all covariates tested
☒	☐	A description of any assumptions or corrections, such as tests of normality and adjustment for multiple comparisons
☐	☒	A full description of the statistical parameters including central tendency (e.g. means) or other basic estimates (e.g. regression coefficient) AND variation (e.g. standard deviation) or associated estimates of uncertainty (e.g. confidence intervals)
☐	☒	For null hypothesis testing, the test statistic (e.g. F, t, r) with confidence intervals, effect sizes, degrees of freedom and P value noted<br>*Give P values as exact values whenever suitable.*
☒	☐	For Bayesian analysis, information on the choice of priors and Markov chain Monte Carlo settings
☒	☐	For hierarchical and complex designs, identification of the appropriate level for tests and full reporting of outcomes
☒	☐	Estimates of effect sizes (e.g. Cohen's d, Pearson's r), indicating how they were calculated

Our web collection on statistics for biologists contains articles on many of the points above.

Software and code

Policy information about availability of computer code

Data collection	ImageJ v 2.0.0 (2018) (Schneider, Rasband and Eliceiri, 2012; https://imagej.nih.gov/ij/download.html), was used to measure the Integrated Fluorescence Density in all the immunohistochemistry assays, as well as to collect data regarding the penetration of Evans Blue and 2-NBDG in control and experimental groups. The rest of the data were obtained without the help of a software.
Data analysis	All the collected data were analyzed using GraphPad Prism Software version 7

For manuscripts utilizing custom algorithms or software that are central to the research but not yet described in published literature, software must be made available to editors and reviewers. We strongly encourage code deposition in a community repository (e.g. GitHub). See the Nature Portfolio guidelines for submitting code & software for further information.

Data

Policy information about availability of data

All manuscripts must include a data availability statement. This statement should provide the following information, where applicable:

- Accession codes, unique identifiers, or web links for publicly available datasets
- A description of any restrictions on data availability
- For clinical datasets or third party data, please ensure that the statement adheres to our policy

Provide your data availability statement here.

Field-specific reporting

Please select the one below that is the best fit for your research. If you are not sure, read the appropriate sections before making your selection.

[x] Life sciences [ ] Behavioural & social sciences [ ] Ecological, evolutionary & environmental sciences

For a reference copy of the document with all sections, see nature.com/documents/nr-reporting-summary-flat.pdf

Life sciences study design

All studies must disclose on these points even when the disclosure is negative.

Sample size	No sample-size calculation was performed.
Data exclusions	Data from no animals were excluded from the present study.
Replication	All attempts at replication were succesfull when the exact same procedure was carried out in different animal groups.
Randomization	Animals were randomly assigned to experimental protocols as well as time points.
Blinding	The analysis of the collected data was a blind process.

Reporting for specific materials, systems and methods

We require information from authors about some types of materials, experimental systems and methods used in many studies. Here, indicate whether each material, system or method listed is relevant to your study. If you are not sure if a list item applies to your research, read the appropriate section before selecting a response.

Materials & experimental systems

n/a	Involved in the study	
[ ]	[x]	Antibodies
[x]	[ ]	Eukaryotic cell lines
[x]	[ ]	Palaeontology and archaeology
[ ]	[x]	Animals and other organisms
[x]	[ ]	Human research participants
[x]	[ ]	Clinical data
[x]	[ ]	Dual use research of concern

Methods

n/a	Involved in the study	
[x]	[ ]	ChIP-seq
[x]	[ ]	Flow cytometry
[x]	[ ]	MRI-based neuroimaging

Antibodies

Antibodies used

Mouse monoclonal IgG anti vimentin Gene Tex, Cat#GTX629744; RRID:AB_2814972.
Rabbit polyclonal IgG anti ZO-1 Thermo Fisher Scientific, Cat#40-2200; RRID:AB_2533456.
Rabbit monoclonal IgG anti GLUT1 Thermo Fisher Scientific, Cat#PA5-16793; RRID:AB_10986893.
Goat polyclonal IgG anti CD31/PECAM R and D Systems, Cat#AF3628; RRID:AB_2161028.
Rabbit polyclonal IgG anti beta catenin Thermo Fisher Scientific, Cat#71-2700; RRID:AB_2533982.
Rabbit polyclonal anti vasopressin, This paper; N/A.
Cy2-AffiniPure Donkey Anti-Rabbit IgG Jackson ImmunoResearch Labs, Cat#711-225-152; RRID:AB_2340612.
Cy3-AffiniPure Donkey Anti-Rabbit IgG Jackson ImmunoResearch Labs Cat#711-165-152; RRID:AB_2307443.
Cy5-AffiniPure Donkey Anti-Goat IgG Jackson ImmunoResearch Labs Cat#705-005-003; RRID:AB_2340384.

Validation

RRID:AB_2814972: validation was supported by the following references
PMID:30377427

RRID:AB_2533456: validation was supported by the following references
PMID:16045494, PMID:17210245, PMID:17846148, PMID:17853450, PMID:18552836, PMID:18757308, PMID:19891526, PMID:20360994, PMID:20552673, PMID:21152390, PMID:21350012, PMID:21429969, PMID:21492152, PMID:21559520, PMID:21617913, PMID:21624488, PMID:21717373, PMID:21852400, PMID:21903671, PMID:21969379, PMID:22287976, PMID:22291907, PMID:22292127, PMID:22327832, PMID:22427845, PMID:22509281, PMID:22705477, PMID:22737109, PMID:22819137, PMID:22895806, PMID:23095816, PMID:23213408, PMID:23351752, PMID:23399832, PMID:23553870, PMID:23621521, PMID:23675413, PMID:23675446, PMID:24075061, PMID:24166498, PMID:24454736, PMID:24505380, PMID:24535401, PMID:24695541, PMID:25003755, PMID:25043620, PMID:25108225, PMID:25187991, PMID:25344575, PMID:25513955, PMID:25540130, PMID:25597427, PMID:25763638, PMID:25894862, PMID:25948584, PMID:26024086, PMID:26109091, PMID:26109581, PMID:26130757, PMID:26182056, PMID:26272951, PMID:26379091, PMID:26411364, PMID:26417608, PMID:26518252, PMID:26678431, PMID:26772200, PMID:26808080, PMID:26829325, PMID:26885895,

PMID:26974005, PMID:26999717, PMID:27008885, PMID:27009841, PMID:27012662, PMID:27023710, PMID:27040283, PMID:27081925, PMID:27097562, PMID:27142375, PMID:27151939, PMID:27167492, PMID:27176222, PMID:27229483, PMID:27278299, PMID:27281269, PMID:27285761, PMID:27381832, PMID:27403660, PMID:27478484, PMID:27605665, PMID:27659202, PMID:28103316, PMID:28117276, PMID:28669519, PMID:28826723, PMID:28950101, PMID:29063836, PMID:29897330, PMID:30269989, PMID:30271583, PMID:30485810, PMID:30824354, PMID:30864945, PMID:30917321, PMID:30932813, PMID:31730175, PMID:32116157.

RRID:AB_10986893: validation was suported by the following references
PMID:18191496, PMID:18402811, PMID:18922940, PMID:19286567, PMID:19603235, PMID:19763184, PMID:21440393, PMID:22153830, PMID:22398711, PMID:22651703, PMID:22819758, PMID:22927057, PMID:23232944, PMID:23392136, PMID:23398456, PMID:23481710, PMID:23485147, PMID:23674658, PMID:23731617, PMID:24134598, PMID:24700751, PMID:25373781, PMID:25830774, PMID:26224287, PMID:26224632, PMID:26284517, PMID:26657503

RRID:AB_2161028: validation was suported by the following references
PMID:28389228, PMID:28882402, PMID:29861385, PMID:29937388, PMID:30269989, PMID:30503141, PMID:30579864, PMID:30639056, PMID:30950036, PMID:31130381, PMID:31543445, PMID:31545012, PMID:31968255, PMID:32073398, PMID:32091396, PMID:32212159

RRID:AB_2533982: validation was suported by the following references
PMID:12533412, PMID:12791303, PMID:15147582, PMID:15880438, PMID:15882576, PMID:18201716, PMID:19224982, PMID:19720156, PMID:20212454, PMID:20396778, PMID:21256972, PMID:21685894, PMID:22252313, PMID:22522917, PMID:22670221, PMID:22797892, PMID:22875787, PMID:23097088, PMID:23248771, PMID:23565266, PMID:24169556, PMID:25217631, PMID:25714812, PMID:25717188, PMID:25777294, PMID:25873562, PMID:25901598, PMID:25976454, PMID:26048141, PMID:26879129, PMID:26888480, PMID:26905250, PMID:27179130, PMID:27358069, PMID:27411103, PMID:28826820, PMID:29434191, PMID:30016153, PMID:30649248, PMID:30808893, PMID:30829292, PMID:30994903, PMID:31157869, PMID:31794718, PMID:31909540, PMID:32513914

Animals and other organisms

Policy information about studies involving animals; ARRIVE guidelines recommended for reporting animal research

Laboratory animals	Young adult male Wistar rats weighing 250–350 g (P50-P65) were used in this study.
Wild animals	This study did not involve wild animals
Field-collected samples	This study did not involve samples collected from the field
Ethics oversight	Experiments were performed following the regulations of ARRIVE (Animal Research: Reporting of In Vivo Experiments) guidelines, the Universidad Nacional Autónoma de México and Instituto de Investigaciones Biomédicas, according to Mexican norms for animal handling (Norma Oficial Mexicana, NOM-062-ZOO-1999) and the law for animal protection published by the University Animal Care Committee in Mexico City, Mex. (February 2002).

Note that full information on the approval of the study protocol must also be provided in the manuscript.
